# The development of connectives in three to five-year-old monolingual Spanish-speaking children

**DOI:** 10.1371/journal.pone.0224461

**Published:** 2019-10-29

**Authors:** Francisco J. Rodríguez-Muñoz, Dimitrinka G. Níkleva

**Affiliations:** 1 Department of Education, University of Almería, Almería, Spain; 2 Department of Language Education, University of Granada, Granada, Spain; Newcastle University Institute for Health and Society, UNITED KINGDOM

## Abstract

Little attention has been paid to the expansion in the use of connectives in children that are acquiring Spanish as a first language. Considering that language structure emerges from language use, naturalistic data were utilised from two conversational corpora. 12 types of connectives showed significant expansion, with the conditional conjunction *si* ‘if’ presenting the greatest advance, followed by *que* ‘that’ used as relative pronoun with explicit antecedent and as completive conjunction. We observed that the interordination and subordination relationships progressed more than those of coordination; however, certain connective elements between coordinated constructions displayed a more notable expansion as discourse markers, in the interactional level.

## Introduction

There is insufficient attention given to the early language development of Spanish as L1 in the Hispanic academic tradition, which has led to sometimes extreme dependence on Anglo-Saxon work; this is even more striking if one considers that Spanish is the language with the largest number of native speakers in the world [1]. The gap in this type of study has been even more noticeable in the field of constructional development as most of the research on Spanish language acquisition has focused on the phonological component from the eighties (e.g., [2]) until more recently (e.g., [3–5]).

When we refer to constructional development, not forgetting that language development is produced in a block, we situate ourselves at the syntactic level and adopt *construction* as the basic unit of analysis, which coincides with the definition of *complex sentence* provided by Diessel [6]; namely, grammatical structures through which specific relationships are expressed between at least two situations in two or more clauses. The term *complex construction* [7] is related to the *constructionist approaches*, for which form and function are indissoluble, as is the language of the cognitive and contextual conditions in which it occurs [8].

This study aims to provide new information regarding the elements (prepositions, conjunctions, relative pronouns and discourse markers) that Spanish-speaking children between three- and five-years old use most frequently to join distinct types of syntactic structures or discursive segments. On another note, we understand that the language structure emanates from its use [7, 9–13], so *grammar-in-process* will be examined through the child’s spontaneous and natural conversation; similarly, the peculiarities of its constructional development will not be described from the *grammatic product* of the adult, rather that the infantile language is conceived as an autonomous and singular model [10]. In summary, it is assumed that grammatical development is epiphenomenal in character and that advances at the constructional level appear together with specific communication contexts [9, 14–17].

### Studies on constructional development in Spanish

In addressing constructional development, three phases associated with specific processes have been distinguished [10, 18]: (1) *predication—*the use of verbal elements and diversification of forms (*tokens*), the increase of which correlates with the index of conversational participation; (2) *combinatorial—*the integration of verbal elements into sequences, which are initially transitive and copulative; and (3) *concordance*—the use of words with grammatical value and relational in character. Although they can be presented sequentially and have a greater predominance at one time or another, the predication stage, which will have a leading role in the child’s initial grammatical development, does not decline when the phase of combination or concordance emerges; in other words, it is necessary to insist on the elasticity of the grammatical acquisition dynamics.

Of the three previous phases, the one of greatest interest in this work is the last, when there is an increase in the use of elements through which syntactic functions are introduced—connectives—above all, from 3 years onwards. Before this age, it is common for structures to be constructed by connecting lexical elements, ignoring the grammatical ones. At the same time, there is the step of repeating the syntactical schemes learned—through the *cutting and pasting* system—to manage their own productive patterns; this process has been described as a transition from a *semantic grammar*, in the initial stages, to a *formal grammar*, whereby the way the words relate to each other contributes to the comprehension of meaning [7, 19, 20].

Regarding the existing data on the development of grammatical complexity in Spanish, it has been pointed out that complex sentences emerge from 2;6 years [21]. Before using coordinating conjunctions, Spanish-speaking children resort to juxtaposed sentences, which, in adult language, one would expect to be linked by a connective [22]—it has even been said that there is a universal order in languages in which juxtaposition precedes the emergence of connectives [23].

Studies that have examined patterns in acquisition of connectives in Spanish have focused on children at ages between 2 and 4 years. The most widely used connectives in Spanish at the age of 2 and a half are the coordinators *y* ‘and’ and *pero* ‘but’. As for subordination, the most frequent class is the relative [24] (for the case of English, see [25]). Although to a lesser extent, the number of incomplete comparative constructions or ellipses is also elevated, and sentences headed by the relative adverb of place (*donde* ‘where’) without express antecedent, and with ellipses. At 4 years of age, only seven connectives are used [26]: *y* ‘and’, *pero* ‘but’, *que* ‘that’, *porque* ‘because’, *cuando* ‘when’, *luego* ‘then’ and *entonces* ‘so’, which are completed at ages between 6 and 11 with *como* ‘like’, *o* ‘or’, *nada más* ‘no more’, *pues* ‘as’, *donde* ‘where’, *despues* ‘after’, *para* ‘for’ and *así que* ‘so’. The most frequent are *y* ‘and’, *que* ‘that’, *luego* ‘then’ and *entonces* ‘then/thus’. As for the types of syntactic relationships, at age 4, coordination with *y* ‘and’ is prevalent, the earliest connective acquired [27], and reduced types of subordination (relative and causal), which will expand from 6 years of age onwards.

### Connective or discourse marker? The syntax-pragmatics interface

In language, there are linking resources that can work in different ways within the same domain, the syntactic or the pragmatic, and at the same time in both. We talk, on the one hand, of conjunctions and prepositions that, as syntactic classes, serve to express relations of meaning between the facts denoted by two sentences, and play a central role in the construction of textual coherence [28]. Discourse markers, on the other hand, operate in the pragmatic domain and are used as expressions of relationships between members or discursive segments, speech acts or turns in conversation. However, as van Dijk points out, these elements must be interpreted with respect to pragmatic contexts in terms of function. Thus, the *semantic use* that is made of sentential connectives must be distinguished from the *pragmatic use* that corresponds to discourse markers [28], as conceived in this study. This distinction between the semantic and the pragmatic use of connectives and discourse markers has been described in terms of the subjectivity of speaker involvement [29]. *Objective relations* are defined as *content or ideational relations* between sentences, while *subjective relations* are identified with *epistemic* and *speech acts relations* [30].

Although the examples provided in (1) and (2) include the same lexical item (*porque* ‘because’) to express a cause-consequence relationship, they do not act on the same domain. In (1) periods are related because of their propositional content, which depends on their locutionary meaning, i.e., a verbal complement is introduced by a subordinating conjunction. On the contrary, in (2) the first segment reflects a reasoning process where a relationship exists between an event and an inference of the speaker, who is clearly involved. The epistemic use of connectives never appears before its logical meaning in the course of language acquisition [31, 32].

(1) CAELO, 5;1
  *CHI: *fue a la casa de su abuelita* ***porque*** *su mamá le dijo que fuera*.
  ‘She went to his granny’s **because** his mom asked her to do it.’
(2) CAELO, 5;7
  *CHI: *ojalá fuera ya verano*, ***porque*** *en verano no hay cole*.
  ‘I wish it were Summer now, **because** in Summer there is no school.’

Some of the elements that we analyse in this study are syntactically polycategorial; for example, *que* ‘that’ in Spanish can be a completive, causal, and even copulative conjunction, while in other sentences, a type of relative pronoun. However, at the same time, *y* ‘and’, *pero* ‘but’ or *pues* ‘as’ belong to the syntactic class of conjunction and, as a result of their decategorisation, they become pragmatic elements, which can carry out additive, counter-argumentative or continuative-type discursive functions, respectively. Therefore, a single unit can belong simultaneously to a syntactic class—conjunctions, prepositions, adverbs, etc. and a pragmatic class—discourse markers [33]. Here, we consider that such elements have undergone a process of pragmaticalisation [34], which determines their use as discourse markers; appropriating, therefore, a “discourse interactional meaning” [35]. The pathway that leads from the first classes to the latter—that is to say, the one that leads to the pragmaticalisation—seems to be the extra-sentential function, from which these units incorporate properties of the discourse markers, such as their mobility in the utterances, their tonicity, the transmission of interpersonal content or the logical-argumentative relations that they carry [36].

In the field that concerns us, one might wonder if there is a comparable evolution between the processes of pragmaticalisation that have been observed in these units within the system and their acquisition in childhood. In other words, are the syntactical functions developed before the pragmatical in the child’s acquisition processes, as is diachronically the case in the linguistic system? Works on language development in English (e.g., [6, 7]) and in Spanish (e.g., [1, 37, 38]) support the idea that these elements firstly unfold pragmatic functions in the interaction, then are later grammaticalized as a syntactic class and perform functions on the sentence plane. This line of research is based on the hypothesis of dependence, support or cohesion with the adult language [39].

### Aims of this study

In view of the lack of research in the Hispanic area addressing early constructional development in children—even more so in the contact area between syntax and pragmatics—more descriptive studies are logically needed to account for the genuine characteristics of children’s language in their different evolutive stages.

In particular, this article aims to determine what types of connectives experience a more notable expansion in conversations between Spanish-speaking children and adults from 3 to 5 years of age, as well as identifying the types of syntactic structures that they connect or introduce most frequently. Therefore, the analysis that we propose will, above all, look at the frequency of occurrence.

Conversely, the most significant advance regarding some of the elements analysed will relate to the functions they play in the interaction; that is to say, we anticipate that, at 5 years of age, several will undergo a more significant expansion as discourse markers at the pragmatic level, beyond merely acting as connectives on the syntactical plane. Hence, this study will also attempt to recognise in which elements such a process is observed.

## Method

### Ethics statement

This study was conducted in accordance with the Declaration of Helsinki and with all the provisions of the human subjects oversight committee guidelines and policies of authors’ institutions (approval code 17-18-1-01C). Ethical permission was granted by the UAL and UGR institutional review boards. Parents and legal guardians provided written informed consent.

### Procedure

In accordance with the premises of the modern observational studies on child language acquisition [40], free conversations between children and adults were tape-recorded in two familiar and everyday situations for the child: the domestic context and at school. For both, the researchers were in charge of collecting the material and motivating, in some cases, the conversations by means of spontaneous questions, game situations, graphic support and stories. To provide the most natural interaction, apart from the researcher, whenever possible, there was one or more interlocutor present from the child’s familiar environment where the recordings were made.

### Corpus

In order to explore the constructional development of children from 3 to 5 years of age, a corpus was collected from a variety of children at the same age, referring to the similar social condition of all the participants and balancing the samples according to the number of conversational turns analysed.

This research is sourced from two corpora of conversational data: (1) BecaCESNo (Benedet and Snow, dirs.), deposited in the international TalkBank database, and (2) CAELO (‘The Corpus of Spanish oral language learners’), in preparation since September 2018. From the first corpus, 2,114 turns were analysed belonging to 7 children (*CHI) aged between 3;4 and 3;9 years, and 2,111 turns from 6 children between 5;2 and 5;7 years of age. From the second corpus, 2,341 turns from 7 children between 3;5 and 3;10 years of age were examined, and 2,943 turns corresponding to 8 children between 5;1 and 5;7 years of age. The conversational turns of the corpus were spoken segments whose final intonation contour was declarative (.), interrogative (?) or exclamatory (!), as well as independent segments that contained a non-final intonation contour indicating continuation (…). The total number of turns of this type was 9,509.

As for the detection of occurrences in the corpus, within the framework of sentence syntax, we considered elements that act as connectives that separated sentence sets or that introduced constructions. Therefore, connectives that headed groups of words that did not form sentences were not computed. In the pragmatic-interactional framework, the discourse markers that appear in the turns have been analysed, irrespective of whether that turn includes verbs. Likewise, the criterion assumed that the element considered as the discourse marker establishes connectives that exceeded the intra- and intersentence limits; that is to say, the relationships are produced, in such cases, between conversational turns, or within the same turn, between discursive segments. In other words, the discourse markers are differentiated from the connectives in which they are neither integrated in the predicate nor externally modify the sentence, but they link the propositional contents with others that carry the preceding discourse [33].

By codifying connectives and discourse markers, the elements that are repeated immediately in the same turn have been dispensed with. In order to guarantee the reliability of the analysis, the coders considered all the contextual information available to interpret the intra and inter-enunciative relationships of the codified units as specifically as possible.

The transcripts were adapted to the standards of the CHILDES (Child Data Exchange System) project from the TalkBank database and the CHAT coding system (Codes for the Human Analysis of Transcripts) [41]. However, 37 labels were developed for the analysed elements, establishing three levels of subcategorization: plane (syntactic = $SYN; pragmatic = $PRA), category (connective = CN; discourse marker = DM) and subcategory (see Table 1). So, for example, the sequence $SYN:CN:CSCJ corresponds to “syntactic level; connective; causal conjunction”, and $PRA:DM:AD, to a “pragmatic level; discourse marker; additive”.

**Table 1:** Coding scheme and examples for syntactic subcategories.

Code	Subcategory	Examples (adult-child interaction, CAELO)
ADCJ	Adversative conjunction	*Mi madre tiene el pelo rubio*, ***pero*** *mi padre lo tiene marrón* ‘My mother’s hair is blonde, **but** my father’s is brown’
CSCJ	Causal conjunction	*Eran amigas* ***porque*** *jugaban juntas* ‘They were friends **because** they played together’
CRCJ	Comparative conjunction	*Su casa era más grande* ***que*** *la de su hermano* ‘His house was bigger **than** his brother’s’
CMCJ	Completive conjunction	*Dijo* ***que*** *iba a venir hoy* ‘She said (**that**) she was coming today’
CCCJ	Concessive conjunction	*No lo sabia*, ***aunque*** *lo había visto* ‘He didn’t know it, **although** he had seen it’
CDCJ	Conditional conjunction	***Si*** *me llevas*, *me lo como todo* ‘**If** you take me, I will eat everything’
CSCJ	Consecutive conjunction	*Era tan pequeño* ***que*** *nadie lo vio* ‘He was so small **that** nobody saw him’
CPCJ	Copulative conjunction	*Tiene un coche* ***y*** *se ha comprado una moto* ‘He has a car **and** he has bought a motorbike’
CCPCJ	Correlative copulative conjunction	***Ni*** *es mi novia* ***ni*** *es mi amiga* ‘She is **neither** my girlfriend, **nor** my friend’
CCDCJ	Correlative disjunctive conjunction	***O*** *me haces caso* ***o*** *no salimos después* ‘**Either** you listen to me **or** we don’t go out afterwards’
DCJ	Disjunctive conjunction	*¿Está aquí* ***o*** *está en su casa*? ‘Is she here **or** is she in her house?’
ECJ	Exceptive conjunction	*Hizo todo*, ***menos*** *lo que tenía que hacer* ‘He did everything, **except** what he had to do’
FPP	Final preposition	*Le gastó una broma* ***para*** *hacerlo reír* ‘He played a joke **to** make him laugh’
ILCJ	Illative conjunction	*No había visto al hombre malo dentro*, ***entonces*** *entró* ‘She hadn’t seen the bad man inside, **then** she went in’
ILCJLC	Illative conjunctive locution	*No sabia si el hombre estaba dentro*, ***así que*** *entró* ‘She didn’t know if the man was inside, **so** she went in’
RAMWA	Relative adverb of mode with explicit antecedent	*Esa es la manera* ***como*** *pasó* ‘That is the way (**how**) it happened’
RAMWOA	Relative adverb of mode without explicit antecedent	*Lo he hecho* ***como*** *me has dicho* ‘I’ve done it **like** you said’
RAPWA	Relative adverb of place with explicit antecedent	*Ponlo en el sitio* ***donde*** *dijimos* ‘Put it in the place **where** we said’
RAPWOA	Relative adverb of place without explicit antecedent	*El libro está* ***donde*** *lo dejaste* ‘The book is **where** you put it’
RAQWOA	Relative adverb of quantity without explicit antecedent	*Dime* ***cuanto*** *sepas sobre la niña de azul* ‘Tell me **how much** you know about the girl in blue’
RATWA	Relative adverb of time with explicit antecedent	*Los días* ***cuando*** *es verano son divertidos* ‘The days **when** it’s summer are fun’
RATWOA	Relative adverb of time without explicit antecedent	*Saldremos* ***cuando*** *acabemos la historia* ‘We will go out **when** we finish the story’
RPPG	Relative prepositional group	*Esa fue la calle* ***en la que*** *vimos a la prima Ana* ‘That was the street **where** we saw cousin Ana’
RPWA	Relative pronoun with explicit antecedent	*La niña* ***que*** *pasea a su perrito no tiene boca* ‘The girl **who** walks her puppy has no mouth’ *Las cosas* ***que*** *hace el niño no están bien* ‘The things (**that**) the boy does are not right’
RPWOA	Relative pronoun without explicit antecedent	***Quien*** *lo sabe es ese hombre* ‘**Who** knows is that man’
RPWOADA	Relative pronoun without explicit antecedent—headed by a definite article	***Los que*** *están en su casa son sus hermanitos* ‘**Those that** are in his house are his little brothers’

In order to determine the degree to which coders 1 and 2 agree when applying these labels to categories and subcategories, inter-observer reliability was determined via percentage agreement (*PA*) and weighted kappa (*κ*) statistics (see Table 2). Results showed that *PA* was over 90% for both coders and values of *k* are good (.60-.75) [42].

**Table 2:** Inter-observer reliability values.

Level of categorization	*PA* (%)	*k*	Strength of values
Category (connective / discourse marker)	96.2	.65	Good
Subcategory	90.4	.61	Good

### Participants

As Table 3 shows, 28 monolingual Spanish-speaking children participated in the study. The mean age of the children in the younger age group was 3;7 (3;4–3;10). The mean age of the children in the older age group was 5;4 (5;1–5;7). The two age groups in our study consisted of equal numbers of boys and girls. According to the information provided by parents on the consent form, none of the participants had any known communicative or neurosensory difficulties, nor were they under any pharmacological treatment at the time of data collection. Therefore, they all manifested typical language development. They also belonged to Spanish families with middle socioeconomic status.

**Table 3:** Demographic information about the participants.

Corpus and linguistic variety represented	Age range	Sex	Key interlocutors
BecaCESNo (European Spanish, central)	3;4–3;7	Female	Mother and another child
		Female	Mother
		Female	Mother and father
	3;4–3;9	Male	Father and aunt
		Male	A boy
		Male	—
		Male	Mother, father, grandmother and another child
	5;2–5;7	Female	—
		Female	Teacher and second researcher
		Female	Aunt
	5;3–5;6	Male	Mother, aunt, uncle and another adult
		Male	—
		Male	Mother, aunt and uncle
CAELO (European Spanish, meridional)	3;5–3;10	Female	Caregiver
		Female	Caregiver
		Female	Caretaker and mother
		Female	Caregiver
		Male	Mother
		Male	Mother
		Male	Mother
	5;1–5;6	Female	Teacher and other children
		Female	Teacher and other children
		Female	Teacher and other children
		Female	Teacher and other children
	5;1–5;7	Male	Mother
		Male	Mother
		Male	Mother
		Male	Mother

### Analytic plan

Statistical analysis was run on R software version 3.1.3 (Bell Laboratories, New Jersey, NJ, USA). The purpose of the analysis was to compare the proportions (*p*) of frequency occurrence (*n*) in relation to the corresponding totals through a descriptive-quantitative and inferential binomial analysis that established the statistical significance of the studied contrasts. As the total of the frequencies was different-not comparable, each frequency was applied to a two-sample *z*-test to differentiate between proportions, *n*_*1*_ and *n*_*2*_ ≥ 5. Also, a Mann-Whitney-Wilcoxon test was performed to check the existence of statistically significant differences between the group of three-year-olds and the five-year-olds. The significance level was set at *p* <. 05.

## Results

According to the *z*-test, of the 26 types of identified syntactic elements that function as connectives in the infantile conversations, 12 showed statistically significant differences (46.2%). Likewise, the one-tailed Mann-Whitney-Wilcoxon test determined significant differences between “three-year-old” and “five-year-old” groups in relation to these connectives (*p* = .032; *U* = 39.5; *z* = -1.85). The critical value of *U* at *p* < .05 was 42; therefore, the result is significant and the alternative hypothesis (*H*_*a*_) is accepted according to which there are differences in the usage frequency of the connectives analysed in both groups. This reveals a greater constructional development at 5 years, which corresponds to the similarly greater presence of 11 types of connectives at this age, since the proportion of relative adverbs of place without express antecedent (*donde* ‘where’) was significantly above that at 3 years of age (see Table 4).

**Table 4:** Connectives between 3 and 5 years of age.

Connective	Three-year-olds *(p*_*1*_*)*	Five-year-olds *(p*_*2*_*)*	Three-year-olds (*n*_*1*_)	Five-year-olds (*n*_*2*_)	*z*-value
*Completive conjunction*	.31	.69	222	492	-10,162
*Copulative conjunction*	.45	.55	288	358	-2,539
*Causal conjunction*	.36	.64	172	310	-6,135
*Adversative conjunction*	.31	.69	82	184	-6,188
*Conditional conjunction*	.10	.90	20	172	-11,288
*Comparative conjunction*	.16	.84	6	32	-4,169
*Illative conjunction*	.11	.89	8	66	-6,659
*Disjunctive conjunction*	.18	.82	6	28	-3,703
*Relative pronoun with explicit antecedent*	.25	.75	106	320	-10,304
*Relative pronoun without explicit antecedent—headed by a definite article*	.17	.83	20	98	-7,161
*Relative adverb of place without explicit antecedent*	.70	.30	38	16	2,929
*Relative adverb of time without explicit antecedent*	.28	.72	64	168	-6,671

*p* < .05

The difference in the frequency of use of the following connectives was not significant: final preposition (*para* ‘for’) (*n*_*1*_ = 132; *n*_*2*_ = 106; *z* = 1.54); relative adverb of place with explicit antecedent (*donde* ‘where’) (*n*_*1*_ = 8; *n*_*2*_ = 6; *z* = .52); relative adverb of mode without explicit antecedent (*como* ‘like’) (*n*_*1*_ = 36; *n*_*2*_ = 36; *z* = .00). The following syntactic units were ruled out by the low frequency of use (*n*_*1*_ or *n*_*2*_ < 5): concessive conjunction (*aunque* ‘although’); illative conjunctive locution (*por lo que* ‘thus’); consecutive conjunction (*tan* … *que* ‘so…that’); exceptive conjunction (*menos* ‘less’); relative pronoun without explicit antecedent (*quien* ‘who’); relative time adverb with explicit antecedent (*cuando* ‘when’); relative adverb of mode with an explicit antecedent (*como* ‘like’); relative adverb of quantity without an explicit antecedent (*cuanto* ‘that’); correlative disjunctive conjunction (*o* … *o* ‘either…or’); correlative copulative conjunction (*ni* … *ni* ‘neither…nor’); and the relative prepositional group (*en el que* ‘in which’).

**Fig 1:**
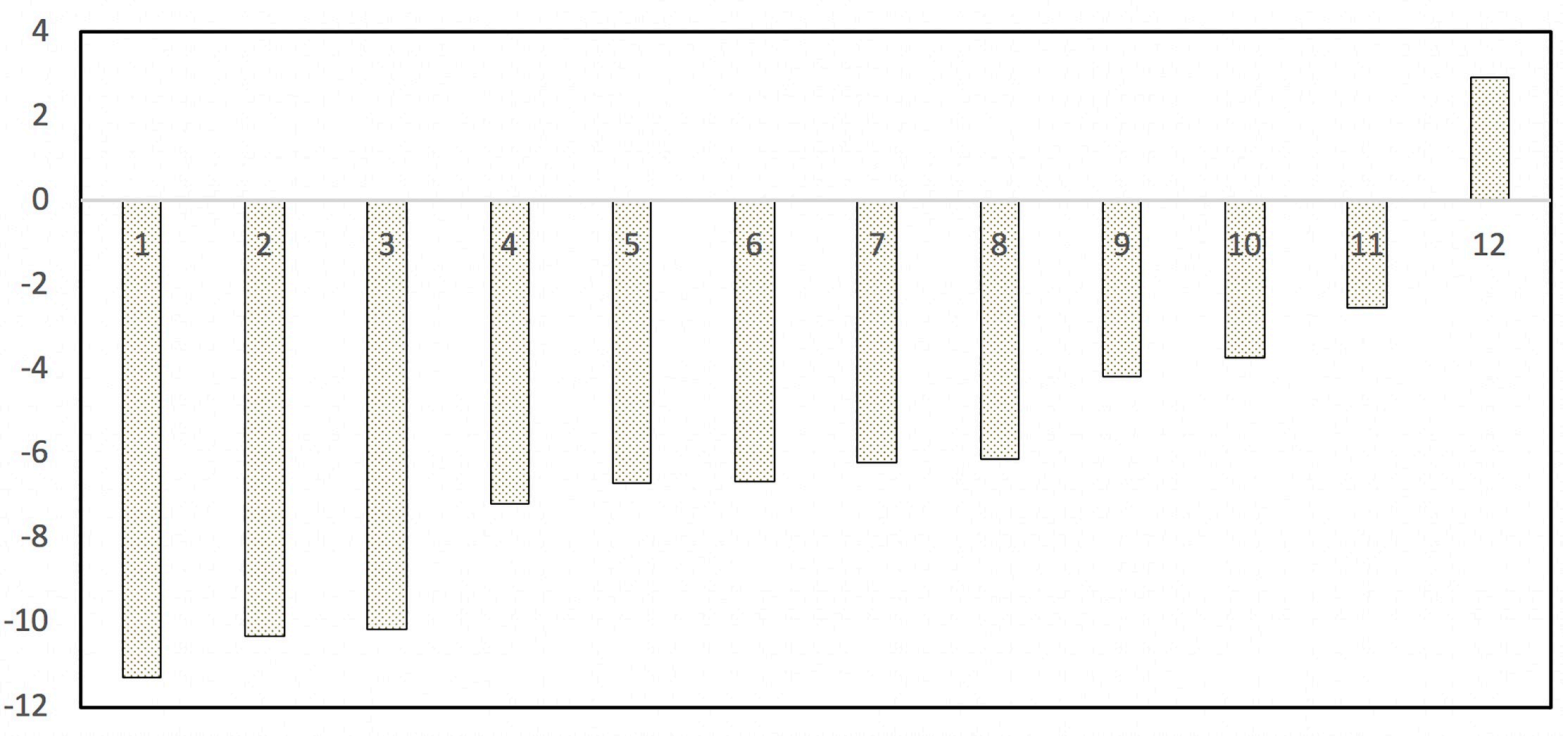
Distribution of the frequency increase of connectives at the constructional level according to their statistical significance. 1. conditional conj.; 2. relative pron. with explicit antecedent; 3. completive conj.; 4. relative pron. preceded by definite article; 5. relative adv. of time without explicit antecedent; 6. illative conj.; 7. adversative conj.; 8. causal conj.; 9. comparative conj.; 10. disjunctive conj.; 11. copulative conj.; 12. relative adv. of place without explicit antecedent.

Based on the *z*-value, in Fig 1 the types of connectives are ordered in terms of their greater to lesser incidence at the constructional level, showing a statistically significant frequency increase in the conversations from three to five-year-olds—except for element 12, which experienced a significant drop in use at age 5.

### Completive and copulative conjunctions

Completive and copulative conjunctions are the syntactic categories most frequently used in children’s conversations at both 3 and 5 years of age. While the first type is a mark of nominal subordination, the second one represents a coordinating conjunction that usually denotes that the next sentence contains additional information. Likewise, the increase in the exploitation of these types of connectives at 5 years of age is statistically significant, even more so for completive conjunction than for copulative conjunctions, the latter being less marked. As for the repertoire of units belonging to these categories, in both cases and at both ages, it is very small. However, the *type-token ratio* is also unable to provide information on the lexical diversity of the speakers, since the Spanish system itself presents a limited inventory for these two syntactic categories; hence, the most common completive conjunction is *que* ‘that’, the copulative, *y* ‘and’—with its contextual variant *e* and negative *ni* ‘nor’.

With regard to the completive conjunction *que* ‘that’, it is very common in the analysed conversations that it is preceded by the verb *ser* ‘to be’ in the third person singular of the present indicative: *es que* ‘is that’—sometimes in the BecaCESNo corpus, this formula is transcribed as *ej que*; this reflects a very characteristic phonetic phenomenon of speech in Madrid, which consists of the aspiration of /s/ before a velar consonant. In these cases, the conjunction serves to introduce a sentence as focal information, emphasizing its relevance or contrasting it with information oriented in the opposite direction [33] and can make structures understood of the type *(lo que pasa/sucede/ocurre) es que…* ‘(what happens/occurs) is that’. Both in these types of copulative constructions as in others, the completive conjunction introduces substantive subordinate sentences in Spanish.

**Fig 2:**
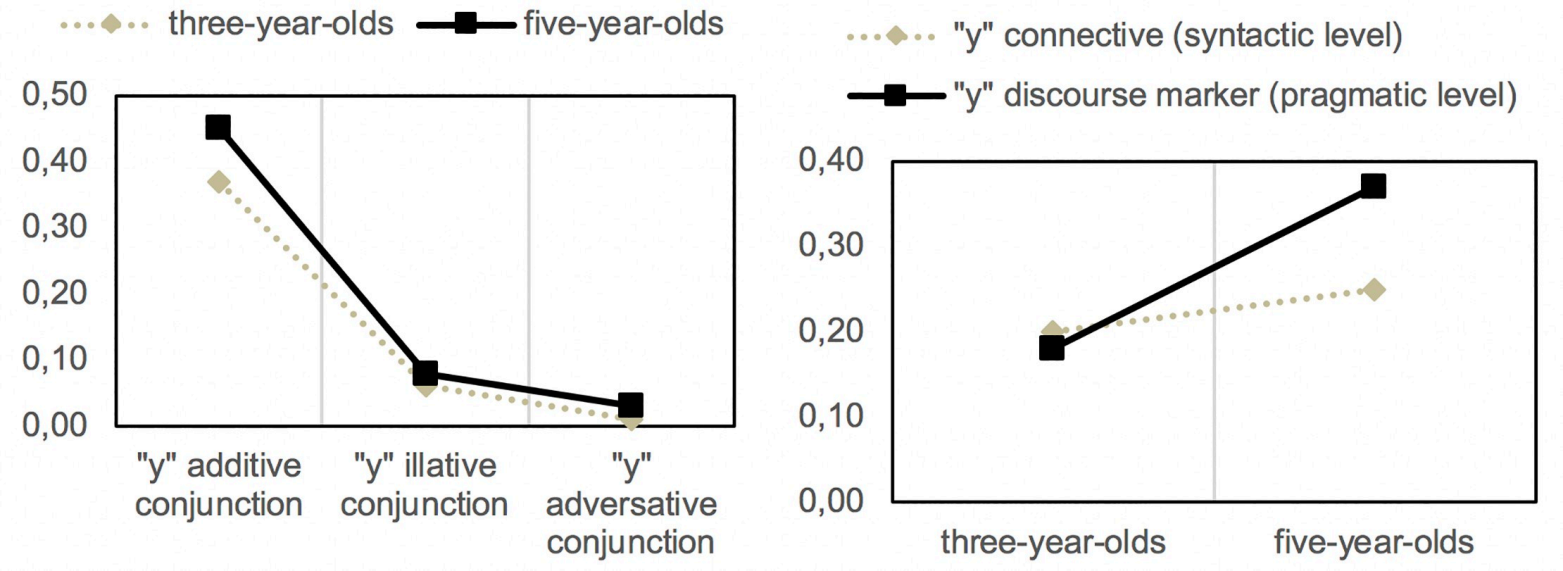
Frequencies of use of *y* with additive, illative and adversative values from 3 to 5 years of age, and its increased use as a discourse marker at 5 years of age.

In contrast, the copulative conjunction *y* ‘and’ serves to unite coordinated sentences; i.e., limbs that do not depend on other syntactic categories, even though it is possible to interpret different nuances, which are distributed in the way observed in Fig 2. In particular, the conjunction *y* ‘and’ supports the following interpretations in the oral samples of three and five-year-olds:

- Additive: The content of the second sentence is in addition to that of the first.
  (3) BecaCESNo, 5;2: *y = (y) además*, *(y) también…* ‘and = (and) as well as, (and) also…’
    *CHI: *a_ver*, *los tiene azules (*.*) es bajo <****y*** *(*.*) tiene el (*.*) pelo entre> [/]* ***y*** *tiene el pelo marrón claro*
    ‘Let’s see, he/she has blue eyes (.) is short <and (.) has the (.) hair between> [/] **and** has light brown hair.’
  Illative: The content of the second sentence expresses a consequence derived from that of the first.
  (4) BecaCESNo, 5;4: *y = por tanto* ‘and = therefore’
    *CHI: *porque no*, *se ponen el abrigo (*.*)* ***y*** *se mojan (*.*) con la lluvia*.
    ‘Because they didn’t put on the coat (.) **and** they get wet (.) in the rain.’
  Adversative: The content of the second sentence contrasts to that of the first.
  (5) CAELO, 5;4: *y = pero* ‘and = but’
  *CHI: *en mi casa no (es)taba (*.*)* ***y*** *en la tuya sí*.
  ‘He/She was not in my house (.) **and** in yours, yes.’

As shown in Fig 2, although at 5 years of age there is an increase in frequency with which *y* ‘and’ is used illatively (*z* = -.37; *p* > .05) and adversatively (*z* = -.37; *p* > .05), statistical analysis determined significant differences only in relation to the additive value of this conjunction from three to five-years of age (*z* = -1.89; *p* < .05). Even so, the most frequent use of *y* ‘and’ is in the absolute initial position of the turns and, in these cases, it is interpreted as a marker that serves to open the discourse, expressing a continuative value equivalent to other connectives such as *pues* ‘as’, (*y*) *entonces* ‘(and) so’, (*y*) *luego* ‘(and) then’, also with an elevated frequency of use in the conversations analysed, in addition to the purely additive (*n*_*1*_ = 266; *n*_*2*_ = 532; *z* = -6.03; *p* < .05):

(6) BecaCESNo, 3;7
  *CHI: ***y*** *le destrozó el país* ***y*** *entonces las casitas volvieron a vivir*.
  ‘**and** tore the country apart and so the cottages lived again.’
  *CHI: ***y*** *entonces colorín colorado (*…*)*.
  ‘**and** so end of story (…).’
(7) BecaCESNo, 5;5
  *CHI: *entonces dijo a Caperucita que dónde iba* ***y*** *entonces ella le dijo que a casa de su abuelita* ***y*** *entonces le dijo el lobo que se fuera por el otro pero […]*.
  ‘So she told Little Riding Hood where she was going **and** then she told her to go to her granny's house **and** then told the Wolf to go for the other but […].’

From these results, it follows that the element *y* ‘and’ is employed at 3 years of age in a proportionally equated manner as a conjunction and as a discourse marker; However, at 5 years of age, there is a very significant increase in its use having an interactional function on the pragmatic plane, if it is compared with its constructional role as a connective on the syntactic plane (*z* = -5.97; *p* < .05).

### Other coordinating conjunctions: Disjunctive, adversative and illative

Although there is a significant increase in the use of disjunctive conjunctions from 3 to 5 year of age (*z* = -3.70; *p* > .05), it is in relation to adversative (*z* = -6.19; *p* > .05) and illative (*z* = -6.66; *p* > .05) conjunctions where the most marked increase is observed. In general terms, disjunctive conjunctions are not usual syntactic categories in the conversations analysed when they act as connectives between coordinated sentences. Nor is there a varied repertoire of conjunctions of this type in Spanish: *o* ‘or’ and its contextual variant *u*.

Similarly, we do not find simple or complex units (locutions) as alternatives to *pero* ‘but’ functioning as adversative coordinating conjunctions—sometimes, at 3 years of age, the phonetic variant *pelo* appears, because the vibrating phonemes are still being acquired. As for illative conjunctions, the inventory is reduced to a single exponent: *pues* ‘as’ as in the case of disjunctive and adversative conjunctions. Still, it should be remembered that *y* ‘and’ assumes the functions of *pues* ‘as’ and, to a lesser extent, *pero* ‘but’ in the discussions examined.

On the other hand, both in the case of the adversative and the illative connectives, analogous pragmatic functions are identified to those performed by discourse markers in oral interactions. The adversative (*pero* ‘but’) and concessive (*aunque* ‘although’) conjunctions find a correlate in the counter-argumentative markers, which operate at the pragmatic level.

**Fig 3:**
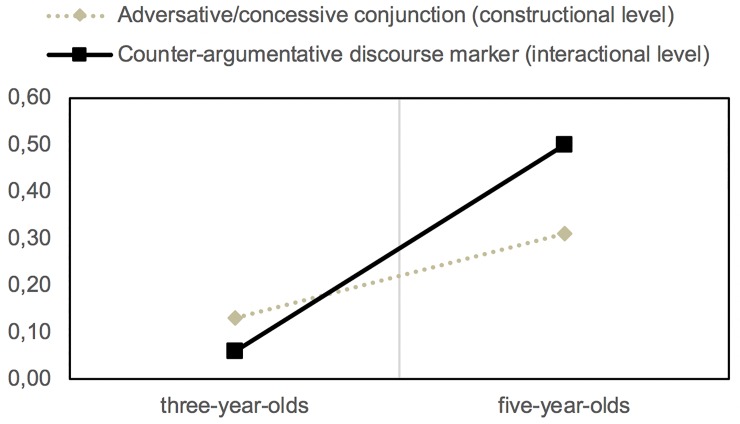
Development of the contrasting expression on the constructional and interactional planes from 3 to 5 years old.

As observed by *y* ‘and’, from 3 to 5 years of age there is a very significant increase in the frequency of use of *pero* ‘but’ with an interactional function; i.e., as a counter-argumentative discourse marker (*n*_*1*_ = 38; *n*_*2*_ = 304; *z* = -14.24; *p* < .05) (Fig 3). If we compare the proportional use of *pero* ‘but’ as a connective and discourse marker at 5 years of age, the differences are equally significant (*z* = -5.35; *p* < .05). In these cases, as in (8), it has interpersonal scope and usually appears in the absolute initial position of the turns, introducing a contrasting movement linked to the immediately preceding turn of an interlocutor.

(8) BecaCESNo, 5;7
  *MAR: *una toallita de limón*, *¿quieres limpia(r)te*?
  ‘A lemon wipe, do you want to clean yourself?’
  ‘*CHI: ***pero*** *¿para qué es*?
  ‘**But** what is it for?’
  *MAR: *para cuando comes gambas en las bodas*.
  ‘For when you eat prawns at weddings.’
  ‘*CHI: ***pero*** *¿para qué es el limón*?
  ‘**But** what's the lemon for?’

A curious phenomenon observed in a few conversations of three-year-olds, as in (9), is that referring to the use of *pero* ‘but’ as *pues* ‘well’; i.e., as a discourse marker on a continuative basis. This may be because *pero* ‘but’ is acquired in isolated cases, before *pues* ‘well’; which would imply it would assume its discursive function.

(9) BecaCESNo, 3;4: *pero* = *pues* ‘but = well’
  *PAT: *¿no has escrito la carta todavía*?
  ‘Haven’t you written the letter yet?’
  *CHI: *<****pero*** *no>*.
  ‘< **but** no >.’

The exceptionality of the previous example is confirmed by the already extraordinary development by the age of 3 of the continuative function on the interactional plane, which also undergoes a significant increase in frequency of use at 5 years of age (*n*_*1*_ = 232; *n*_*2*_ = 444; *z* = -8.34; *p* < .05). When comparing the proportional use of *pues* ‘well’ with the syntactic value in the constructional plane, and that corresponding to its pragmatic value in the interactional plane, we realize that, at 5 years of age, there is an absolute prevalence of the second type (*z* = -16.68; *p* < .05). This function is mainly carried out by the discourse markers of *y* ‘and’, *pues* ‘as’, *entonces* ‘so’ and *luego* ‘then’.

**Fig 4:**
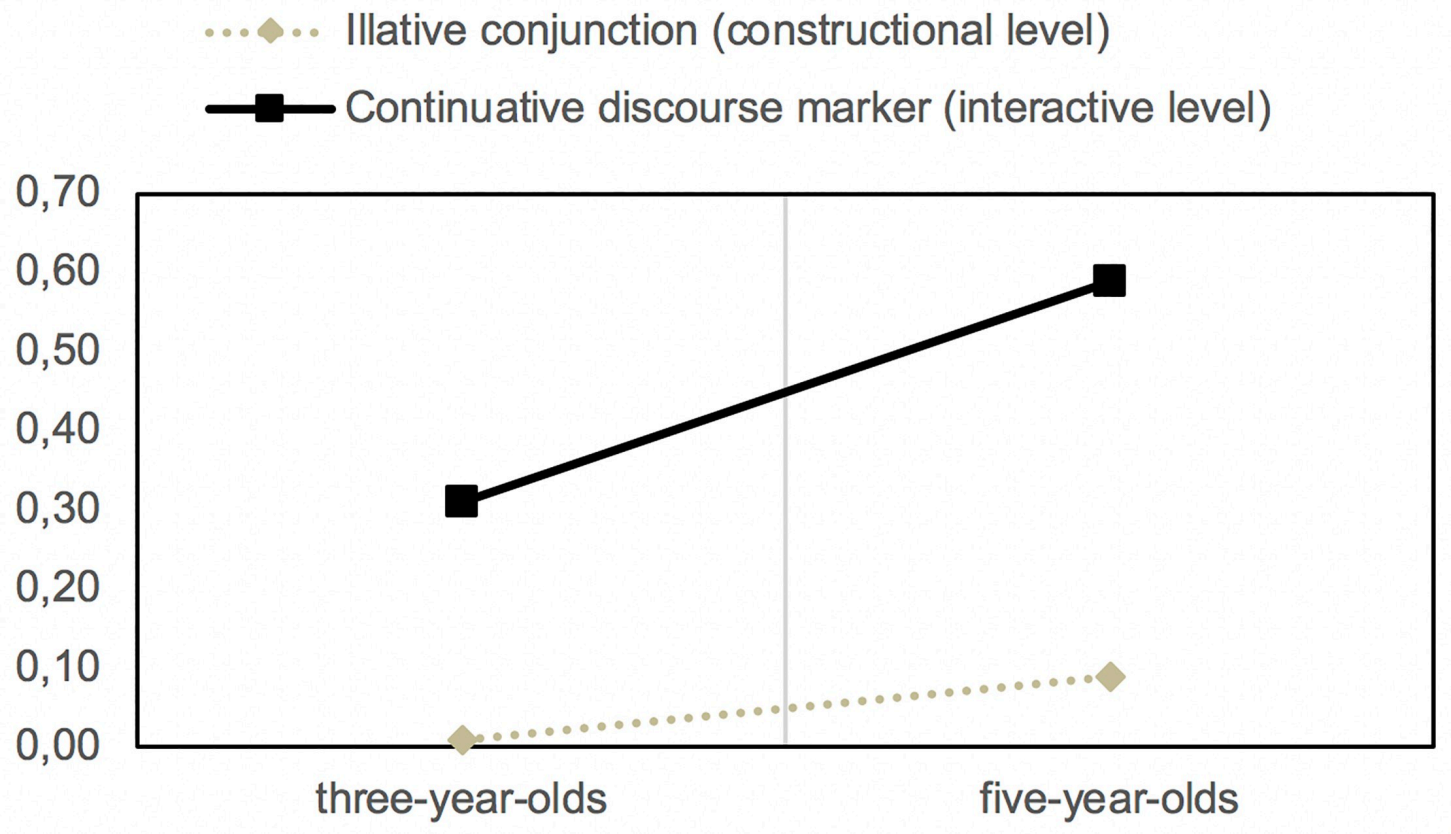
Development of the expression of illation/continuity on the constructional and interactional planes from 3 to 5 years of age.

As Fig 4 shows, the early development of *pues* ‘as’ with a discursive function contrasts with its low productivity on the constructional plane at 3 and 5 years of age, as an illative conjunction that, in our conversations, does not alternate with any other exponent—only the phonetic variants *pos* and *p(u)es* are recorded.

### Other subordinate conjunctions: conditional, causal and comparative

According to our data, there is significant development from 3 to 5 years of age at the constructional level to establish conditional (*z* = -11.29), causal (*z* = -6.14) and comparative (*z* = -4.39) relationships, in that order, using subordinate conjunctions in conversations.

The most characteristic connective that heads conditional constructions in Spanish is *si* ‘if’, as in the oral interactions we analysed. In these cases, on the one hand, the conditional clause (*protasis*) often precedes the conditioned clause (*apodosis*) and, on the other, other particles appear (*y* ‘and’, *o* ‘or’, *pero* ‘but’, *porque* ‘because’, *pues* ‘as’) before the connective *si* ‘if’.

The connective that typically introduces causal constructions in Spanish coincides with the most common in the conversations analysed: *porque* ‘because’—with phonetic variants: *po(r)que*, *polque*—. As for the repertoire of causal connectives, together with *porque*, at 3 years of age *que* ‘that’ (24.42%), *como* ‘like’ (4.65%) and *pues* ‘as’ (1.16%) are used, while at 5 years of age, *como* ‘like’ (23.23%) and *que* ‘that’ (7.1%). Through the conjunctions that alternate with *porque* ‘because’, external causals to the verbal predicate are usually constructed, also called explicit causals as they often provide a justification that the speaker infers about the context; i.e., the cause from which a conclusion is expressed about something that is not found in that utterance but in the enunciation.

Causals headed by *que* ‘because’ and *pues* ‘as’ are always postponed, whilst those headed by *como* ‘like’ come before; moreover, they are all external to the predicate and explicit in character. In terms of informativity, in the former, the information is shown as an unfamiliar justification (10), whereas in the latter, the information is presented as if it were already known (11). Apart from this, the causals introduced by *que* ‘because’ tend to be preceded by an imperative (for related theory with English adverbials, see [43] and [44]).

(10) BecaCESNo, 3;7
  *CHI: *cuéntamelo tú (*.*)* ***que*** *yo no sé las letras*.
  ‘You tell me (.) **because** I don't know the letters.’
(11) BecaCESNo, 3;4
  *CHI: ***como*** *se han monta(d)o to(do)s (*.) han tenido que segá [*] la calle y se han queda(d)o en la calle.
  *%err*: *segá = cerrar $PHO*
  ‘As they have put up everything (.) they have had to close [*] the street and have stayed in the street.’

*Porque* ‘because’ is used to introduce both internal and external causalities to the predicate, and the causal constructions headed by that connective may either come before or after; despite this, in the samples, this element usually occupies the initial margin of the children's reactive turns, as in (12), and serves to answer questions with *por que* ‘why’, something that does not allow external causals.

(12) BecaCESNo, 5;2
  *INT: *¿por qué*?
  ‘Why?’
  *CHI: ***porque*** *me llaman Monicaca*.
  ‘**Because** they call me Monicaca.’

Finally, we look at the constructions that establish quantitative relationships. These are classified according to the quantifiers that form them in a) comparatives of superiority, b) comparatives of equality, and c) comparatives of inferiority. In these constructions, we distinguish between quantitative groups and comparative complements introduced by *que* ‘that’ and *como* ‘like’. At 3 years of age, these constructions are very unusual in the samples analysed; of the 6 that we identify, there are equality (*tan* … *como* ‘so… that’: 50%) and superiority (*más* … *que* ‘more…than’; *mayor que* ‘greater than’: 50%). In (13) *mayor* ‘greater’ does not function as a comparative but is considered an adjective in a positive degree since it is combined with the *más* ‘more’ quantifier.

(13) BecaCESNo, 3;4
  *CHI: ***más*** *mayo(r)* ***que*** *yo*.
  ‘Older than me.’

The increase in the frequency of use of these types of constructions at 5 years of age is significant; they are distributed as follows:

- Superiority comparatives (53.1%): *más… que* ‘more than’.
- Equality comparatives (34.4%): *lo mismo que* ‘the same as’, *el mismo que* ‘the same as’ and *igual que* ‘the same as’.
- Inferiority comparatives (12.5%): *menos* … *que* ‘less … than’.

Also, at the age of 5, there are ponderative consecutive constructions that are absent in the conversations of three-year-old children, whose outline is analogous to that of the comparatives. In all the examples that we find, as in (14) and (15), there is a repeat of the quantifying group, either by the determinant that heads it, or of the whole group; from a pragmatic perspective, this implies a double intensification of that utterance because it is self-ponderative (indicating that an extreme degree of something has been achieved) and is reinforced by repetition:

(14) BecaCESNo, 5;7
  *CHI: *había una vez un zapatero* ***tan*** *pobre (*.*)* ***tan*** *pobre (*.*)* ***que*** *no podía comprar más cuero para hacer zapatos*.
  ‘There once was a shoemaker **so** poor (.) **so** poor (.) **that** he couldn’t buy more leather to make shoes.’
(15) BecaCESNo, 5;8
  *CHI: *este era un hombre* ***tan*** *(*.*)* ***tan*** *(*.*)* ***tan*** *(*.*)* ***tan*** *gordo (*.*)* ***que*** su &esto estómago le llegaba hasta lios [*] pies.
  *%err*: *lios = los*
  ‘This man was **so** (.) **so** (.) **so** (.) **so** fat (.) **that** his &this stomach touched his [*] feet.’

### Relative pronouns and adverbs

Of all the connectives that introduce relative sentences in Spanish, there are only 4 that significantly progress from 3 to 5 years of age; namely: 1) the relative pronoun with explicit antecedent; 2) the relative pronoun without explicit antecedent headed by the definite article; 3) the relative adverb of place without explicit antecedent; and 4) the relative adverb of time without explicit antecedent.

In all cases, they introduce subordinate sentences; those headed by a relative pronoun with an explicit or incorporated antecedent, as in (16) and may act as a noun complement; conversely, those that do not have an explicit antecedent but are headed by a definite article, also called semi-free relatives, can perform various functions, such as being the subject (17) or direct complement (18).

(16) BecaCESNo, 3;7
  *CHI: *era una vez una barquita de una enanita* ***que*** *vivía en un país muy lejano con los reyes [*…*]*
  ‘There was once a small boat of a dwarf **that** lived in a very distant country with the king and queen […].’
(17) BecaCESNo, 5;4
  *CHI: *por ejemplo (*.*)* ***las que*** *se visten de monjas (*.*) hacen miedo a todas las niñas (*.*) y eso tampoco*.
  ‘For example (.) **those who** dress up as nuns (.) scare all the girls (.) and not that either.’
(18) BecaCESNo, 3;6
  *CHI: *<el cuando> [//] ya sé* ***lo que*** quieres hacel [*] para entlal [*] en mi casa quieres comelme [*].
  *%err*: *hacel = hacer $PHO; entlal = entrar $PHO; comelme = comerme $PHO*
  ‘When I know **what** you want to do [*] to enter my house, you want to eat me [*].’

Relative subordinates that have no explicit antecedents, also called *free relatives* can be introduced both by pronouns and relative adverbs. Those showing a significant expansion in the conversations we analyzed from 3 to 5 years of age are only those introduced by *cuando* ‘when’, and function as an adjunct or time circumstantial (19); thus, when the relative adverb *donde* ‘where’ introduces a clause functioning as an adjunct or place circumstantial, as in (20), instead of observing greater exploitation of these types of structures at 5 years of age, a significant drop in use occurs compared to at 3 years of age:

(19) BecaCESNo, 5;8
  *CHI: *yo me cabreo* ***cuando*** *las cosas no se pueden hacer*.
  ‘I get angry **when** things can't be done.’
(20) CAELO, 3;5
  *CHI: *y vino uno* ***donde*** *estaba yo*.
  ‘And one came **where** I was.’

## Discussion

### Interordination and subordination vs. coordination from 3 to 5 years of age

In contrast to subordinate conjunctions, such as the completive *que* ‘that’ which introduces subordinate clauses dependent on another syntactic category that they complement or modify, the coordinative conjunctions, such as the copulative *y*, unite independent sentence conjuncts which do not depend on other categories. Generally speaking, the types of sentences that advance or expand more from 3 to 5 years of age are those that are associated with interordination and subordination. However, it is advisable to note certain nuances regarding these types of structures present quite different properties amongst themselves:

- Conditional constructions, typically introduced by the conjunction *si* ‘if’, are those that experience the most significant frequency increase at 5 years of age. These are major (regular) structures that do not modify the predicate; instead the two periods that integrate them (conditioning and conditioned) maintain an interdependent relationship (or interordination) so the conditional constructions are considered sentence complements, external or peripheral.
- Sentences headed by relative pronouns with an explicit antecedent (*que* ‘that’), which are the second type whose frequency increases more significantly at 5 years of age; they are subordinates that modify the preceding segment (antecedent), with which they maintain an anaphoric relationship, so that the relative exerts a subordinate connection when introducing a clause as a dependent sentence. Relative semi-free sentences are also considered subordinate, those that are headed by the definite article plus a relative pronoun—they are the fourth whose frequency increases most significantly at 5 years of age.
- Sentences introduced by a completive conjunction (*que* ‘that’), the third type that progresses most significantly at 5 years of age in terms of frequency of use in conversation. These are also subordinate (substantive, completive or declarative) and they perform the same functions as nouns or nominal groups.

Only starting from the sixth position out of the twelve we have established coordinating conjunctions appear; their use, as in the case of subordinates, increasing significantly in statistical frequency at 5 years of age: the illatives—analyzed by some grammarians as subordinate links [see 33]—the adversatives, the disjunctives and, finally, the copulatives. Also, in the second sextile are those conjunctions that head causal constructions, which, as in the case of conditionals, are sometimes interpreted as peripheral complements—this is the case for the external causals—although at other times, they can function as adjuncts or circumstantials—when the causal sentence is internal to the predicate and subordinate to it.

### Constructional development in Spanish linked to the expansion of connectives from 3 to 5 years of age

After analysing the 12 connectives in the oral interactions of the 28 children, the syntactic category found to advance most from 3 to 5 years of age is the conjunction *si* ‘if’, which appears in major sentences structures headed by conditional constructions.

With regard to the relative structures (S1-*wh*-S2), it has been pointed out that the function that first develops the *wh*-forms in complex sentences is *complementation* (i.e., a relative sentence without an antecedent), whereas the function of *relativization* (i.e., a relative sentence with an antecedent) would be posterior [45]. According to our data, at 3 years of age, there is a similar trend in Spanish; i.e., relative sentences without an explicit antecedent (*n* = 160) are proportionally higher than those constructed with an antecedent (*n* = 118) (*z* = -2.67; p < .05). However, the analysis of spontaneous conversation samples reveals a very notable expansion of relative sentences with an explicit antecedent (*relativization*) from 3 to 5 years of age, the second syntactic category in terms of advancing most at these ages.

On the other hand, the first two types of sentences used by children in Spanish are the direct complement subordinates without a connective (*Quiero ir al parque* ‘I want to go to the park’) and the coordinatives with *y* ‘and’ [21]. The relative subordinates, of which we have already mentioned the causal, the temporal and the direct complements introduced by *que* that appear later, between 2 and 3 years of age; however, the development of the latter is not completed until the age of 5 [46]. According to our data, the completive conjunction is the third syntactic category that experiences the most expansion from 3 to 5 years of age, although it does not only introduce direct complement subordinate sentences.

Referring ourselves to the acquisition of English as a first language, the results of our analysis of *y* coincide with that of Bloom et al. [45], where the first syntactic connective produced by children from 2 to 3 years of age is *and*, the equivalent of *y* in Spanish. Taking into account the polyfunctional character of this syntactic-pragmatic element, they establish the following sequence for the emergence of the semantic values corresponding to those ages: 1) additive, 2) temporal, 3) causal, and 4) adversative. In the interactions analysed, the additive value of the copulative conjunction also predominates, followed by the illative conjunction, which relates to the temporal value of posteriority and to consequence—and lastly an adversative value is attributed.

For Peterson and McCabe [47], *y* is not initially an “all-purpose connective” that progressively gives way to other more specific connectives through which the same semantic relationships are expressed by the copulative conjunction. However, in our analysis, this intuition is confirmed to some extent. So, while *y* ‘and’ is the connective that experiences the least expansion from 3 to 5 years of age of all those analysed, the increase in the use of the adversative conjunction *pero* ‘but’ and the illative *pues* ‘as’ is proportionally greater, despite the fact that, as noted above, the use of *y* ‘and’ also increases. Both the results of the naturalistic and the experimental studies correspond in that the additive relationships are acquired before the causal ones and, consequently, the additive connectives emerge before the causal connectives [48].

With regard to *pues* ‘as’, in the samples examined by Aguado [24] and Idiázabal [49] at 2;6 years and between 1;11 and 3;2 years, respectively, no use is recorded. Enríquez [1] dates the emergence of *pues* at 3;1 years, but only records its use up until 4 years of age as a discourse marker, not as a conjunction. Lastly, it has been pointed out that the subordinate conjunction *porque* ‘because’, linked to the causal expression, is not fully controlled until 4 years of age [23]. *Porque* ‘because’ functions more like “a wild card” or “a form of responding” at 2 and a half years than as the introducing connective of a subordinate [24]; in fact, in the interactions analysed, it tends to act as a marginal element that heads reactive turns. In other words, its production is not usually spontaneous, rather the adult gives the child the pattern in the interactions by explicitly asking something using the sequence *por que* ‘why’, which would function as a key or discursive guideline determining the use of *porque* ‘because’. The truly causal relationships that are produced spontaneously by the child have been identified in Spanish from the age of 6 [26]. Nonetheless, in our analysis, *porque* ‘because’ undergoes a fairly significant expansion from 3 to 5 years of age.

### Syntactic function vs. pragmatic function: the transcategorical elements

As has been demonstrated, some of the elements analysed that behave as connectives in the constructional level, and experience a notable expansion from 3 to 5 years of age, also increase their interactional functions in a very significant way at 5 years of age—*y*, which acts as an additive-continuative discourse marker; *pero* ‘but’, which assumes counter-argumentative functions in the conversation; and *pues* ‘as’, which has a continuative role equivalent to that of adverbs that are pragmaticalized in the same way, such as *entonces* ‘so’ or *luego* ‘then’, with the special presence in narrative sequences of autobiographical episodes based on experiences and personal memories, and in others pertaining to learnt classic stories that are reproduced in conversations (e.g., *The three little piggies*, *Little Red Riding Hood*, etc.).

The frequent combination of *y* ‘and’ as a discourse marker with other markers such as *entonces* ‘so’ or *luego* ‘then’ is also commented on in relation to *and*, which may appear along with *then (and then* …*)* [45]. The use of the discourse marker with an additive-continuative value is recurrent even within the same turn and is associated primarily with narrative sequences; this recursion of the marker has been considered a characteristic of child language and has been interpreted as a pragmatic intensification strategy [26]. The use of *pues* ‘as’ on the interactional plane also coincides often with the additive-continuative corresponding to *y* ‘and’. This type of *pues* ‘well’ has been labelled as a commentator, a discursive starter marker and an explanation [1].

As for *pero* ‘but’, our results confirm those of Varela [50] in children aged between 2 and 4 years, who established its origin in the interaction; i.e., its use in infantile speech emanates from an essentially dialogic process and from collaborative co-construction with the adult. Thus, this often corresponds to a responsive function that logically transcends the sentence plane: following the turn initiated by an adult interlocutor, the child reacts to what was said using the counter-argumentative marker *pero* ‘but’, which introduces a contrasting movement in the form of objection.

On the other hand, although this study has analysed the sequence *es que* ‘is that’ as the combination of the copulative verb immobilized in the third person (*es* ‘is’) and the completive conjunction (*que* ‘that’) [33], other authors have considered that the formula is fixed and pragmaticalized; therefore, at the argumentative level, it would constitute a justification-type discourse marker [51], which would require the presence of a previous utterance. Likewise, at the informative level, it acts as a focuser while, in other cases, as an intensification mechanism [52].

When pragmatic functions are identified for the analysed elements; that is to say, whenever they are considered discursivised, these are usually located at the initial margin of the turns, either preceded by some other particle or in the absolute initial position—in other cases, as when *y* acts as an additive-continuative in narrative sequences, it may appear after a pause in the same turn. This increase in frequency of pragmatic functions is particularly marked at the age of 5 when using *pues* ‘well’ in oral interactions.

In summary, those elements that can carry out both syntactic and pragmatic functions, which we have called transcategorial, show more pronounced development of the latter function between 3 and 5 years of age, although there are also examples recorded in which they act as coordinative conjunctions, in the case of *y* ‘and’, *pero* ‘but’ and *pues* ‘as’, or subordinates, in the case of *que* ‘that’. However, they are usually present in the absolute initial position of the turns and establish logical-argumentative relationships that are additive(-continuative) (*y* ‘and’), counter-argumentative (*pero* ‘but’) and continuative (*pues* ‘as’) in type, on the one hand; and justificative (*es que* ‘is that’), on the other. Therefore, our results support the hypothesis of dependence with the adult language [39] and are aligned with those of other authors for whom the pragmatic functions of these transcategorical elements precede the syntactic functions, which are deployed *a posteriori* in the acquisition process [1, 37, 38, 50]. In other words, in contrast to the tendency observed diachronically in the linguistic system, where first the syntactic functions develop and then extrasententially one arrives at the pragmatic functions, in the development of the language, the child firstly resorts to these elements for interactive and interpersonal purposes, and then gives them grammatical value—the child grammaticalizes them. Therefore, the pragmatic-discursive functionality precedes the purely constructional.

## Conclusion

From the analysis of the children’s spontaneous speech, the present study has dealt with expansion rather than emergence (as is more usual in the bibliography) of connectives from 3 to 5 years of age in Spanish. Accordingly, it has been shown that the expression of conditionality using bipolar constructions headed by *si* ‘if’ is the one showing the greatest expansion, followed by the relative *que* ‘that’ with antecedent and the completive conjunction *que* ‘that’. Therefore, it has been verified that, from 3 to 5 years of age, the interdependent (or interordinational) and dependent (or subordinate) relationships between structures increase.

It has also been shown that those transcategorical elements capable of operating simultaneously on two planes, the syntactic and the pragmatic, such as *y* ‘and’, *pero* ‘but’ or *pues* ‘as’, continue to show greater productivity at the interactional level, such as discourse markers, than at the purely grammatical level. As has been pointed out, before being used as conjunctions, they correspond to interactional functions, based on items and patterns learnt in a specific context that can later be imitated and reproduced, which the child will have to recover and extrapolate to comparable communicative situations.

Finally, in the future, it would be advisable to more profoundly investigate the syntagmatic-discursive dynamics in which such elements are produced as those analysed here; for example, after initial labelling, to which this work gives account, it would be possible to establish the relationships between the codified units and the position that they occupy in the utterances, which can be linked to specific informative functions such as focalisation, or to certain types of turns (initiative, reactive, etc.) or speech acts, amongst other things. Likewise, it seems necessary to more profoundly investigate, along with others, development during the infantile period of those elements in Spanish that are adverbial in character, which can act as discourse markers and as modalizers of various kinds on the interactional plane.

## Supporting information

S1 Appendix CHAT codes. (PDF)
