# Supplementary material for: The development of connectives in three to five-year-old monolingual Spanish-speaking children
Source: PLoS One. 2019 Oct 29;14(10):e0224461. doi: 10.1371/journal.pone.0224461 (PMC6818806; doi:10.1371/journal.pone.0224461)
Supplement: S1 Appendix — (PDF) [file pone.0224461.s001.pdf]

# Appendix. CHAT codes

| Symbol | Description |
|---|---|
| *CHI | ID header for each participant (in this case, “child”) |
| (.) | Short pause between words |
| [/] | Repetition: retracing without correction |
| [//] | Retracing: self-corrections or changes |
| <text> | Material being retraced |
| (es)taba, limpia(r)te | Shortenings and sound omissions |
| [*] | Error marking: the form actually produced is placed on the main line and the target form is given on the %err line |
| & | Phonological fragment: incomplete expression |
